# Reproducibility and research integrity: the role of scientists and institutions

**DOI:** 10.1186/s13104-021-05875-3

**Published:** 2021-12-14

**Authors:** Patrick Diaba-Nuhoho, Michael Amponsah-Offeh

**Affiliations:** 1 Division of Vascular Endothelium and Microcirculation, Department of Medicine III, University of Technology Dresden, Fetscherstr. 74, 01307 Dresden, Germany; 2 Institute of Physiology, Faculty of Medicine, University of Technology Dresden, Fetscherstr. 74, 01307 Dresden, Germany

**Keywords:** Research integrity, Reproducibility, Research scientists, Research institutions, Stakeholders, Individual contributions

## Abstract

Reproducibility and research integrity are essential tenets of every scientific study and discovery. They serve as proof that an established and documented work can be verified, repeated, and reproduced. New knowledge in the biomedical science is built on the shoulders of established and proven principles. Thus, scientists must be able to trust and build on the knowledge of their colleagues. Scientific innovation and research discoveries especially in the field of medicine has contributed to improving the lives of patients and increasing life expectancies. However, the growing concerns of failure to comply with good scientific principles has resulted in issues with research integrity and reproducibility. Poor reproducibility and integrity, therefore, may lead to ineffective interventions and applications. Here we comment on research reproducibility in basic medical and life sciences with regards to issues arising and outline the role of stakeholders such as research institutions and their employees in addressing this crisis.

## Introduction

Research integrity encompasses all the (un)known steps in establishing research from the proposal, performance, and evaluation of research as well as the reporting of results that comply with the accepted professional codes and norms. Additionally, it ensures honesty, accuracy, efficiency and objectivity in the scientific research [6]. The reproducibility, integrity and quality of research plays critical role in not only enforcing and protecting good scientific policies and guidelines, but also safeguarding public confidence in research outcomes. However, the fundamental principle of scientific integrity and trustworthy research is the ability to reproduce initial scientific outcomes and findings as well as the consistencies in similar research findings from different groups [2]. Although, the lack of reproducibility does not necessarily indicate scientific misconduct, reproducibility promotes trust, ensures credibility and reliability of research outcomes [5]. Hence, a lack of it can destroy science resulting in mistrust between scientists as well as public confidence in scientific findings and data. This commentary delves into research reproducibility particularly in the life and medical sciences.

## Main text

### Scope and extent of reproducibility

It will be a daunting task to define the exact components of how a reproducible research should be designed especially in the life and medical sciences. This is because of the great deal of variability in biological systems and the complex techniques employed in the design and execution of such studies. Although, it is not essential and expected to reproduce precise results, at least the major findings and underlining conclusions from a research must be potentially validated from similar studies. Therefore, reproducibility is more or less the ability to draw similar conclusions from replicates studies. Also, in our field, reproducibility can be described as taking an existing dataset from a study, re-running the same analysis strategy that was used, and hopefully producing the exact same statistical findings, this is usually useful for spotting errors. On the other hand, replication is utilising the same or very similar methods as an existing study to collect new data, analysing it, and producing the same pattern of results to draw the same overall conclusions [8]. However, the last couple of decades have revealed shockingly the irreproducibility of several studies to validate their major conclusions. This was further heightened by the inability of original authors to reproduce their own experiments [2]. The manifestation of the reproducibility crisis became more visible in clinical trials by the increasing failure of novel treatment strategies, which were efficacious in diseased animal models [7]. A recent survey revealed that at least 70% of scientists were unable to reproduce studies from other scientists as well as the inability of at least 50% of researchers to reproduce their own work [1]. The increasing concerns of this reproducibility crisis has triggered the implementation of policies and guidelines to safeguard research credibility and trustworthiness. Therefore, in order to provide solutions, it is important to identify the root causes of the crisis.

### Factors that influence the reproducibility crisis

The scientific research is a complex process involving several stakeholders at multiple steps such as research design, ethics and legal framework, funding, methods, documentation, publication and archival of research findings. Moreover, many scientists and research hope to reveal or identify novel findings and therapies [5]. Hence, multiple factors contribute to the irreproducibility of a study. These include but not limited to, inadequate training of researchers in experimental design and methodology such as randomization, bias, replication, statistical analysis, variations in sophisticated medical techniques that are difficult to replicate, and variability in chemicals and reagents especially in experiments involving the use of antibodies. Additionally, the insufficient amount of time used for research, the bureaucracy and pressure to publish in high impact journals to compete for research grants and positions as well as lack of proper supervision and mentorship further exacerbates the reproducibility crisis [1, 3]. These may lead to researchers taking shortcuts, not transparently reporting their work, or even using questionable research practices.

### Addressing the reproducibility crisis

Researchers are at the forefront of innovative findings. The same have being at the centre of recent scientific misconduct in different scientific fields [4]. Although researchers and scientists play a dominant role in the research process, collaboration with multiple stakeholders such as academics, regulators, publishers, institutions, funders, and government are required to address the multifaceted reproducibility crisis. Moreover, the recent pandemic has exposed these issues, which indeed should be the foundation of science. We outline some suggestive remedies to this crisis by addressing the specific role of researchers and institutions.

First-off, researchers should be more open to share ideas, methods and data with thematic colleagues and the public. Researchers should devote more time for careful planning, design and execution of scientific research including the use of appropriate experimental methods and statistical analysis, which are necessary to arrive at a good and a reproducible research outcome. Though most researchers would probably like to do these things, they feel overburdened to the point that systemic pressures and misplaced incentives prevent them from doing these things. Research group leaders and supervisors must provide adequate supervision, mentorship, and training to early career researchers to design good experiments from the onset. All research authors must be able to provide the raw data used in their study and should be made accessible to everyone without barriers. Possibly setting up and making accessible data repositories for published papers will allow for transparency and integrity in the research arena. Primary data is very crucial in research findings, hence avenues to store and avoid manipulation is essential. One way to ensure reproducibility of results is to have a clear and concise documentation. This could be done electronically or manually as in safe book-keeping of research data and findings which are openly accessible. Documentation could include open workflows, registered study protocols and methodology. These are important since documentation can be misrepresented if not accessible as part of shortcuts and poor research practices, thus maintaining the crisis. Within research groups, group leaders or supervisors must recheck storage of experimental results and when in doubt, experiments must be repeated by individuals or others in the laboratory to confirm breakthrough findings. For instance, in data analyses, at least two researchers should be tasked to do same analyses. In ensuring the completeness of data in the context of publication, scientists and journal editors must ensure that data should be contextualized instead of over generalized. Since good data also depends on proper laboratory management, established and standardized protocols as well as good and calibrated equipment with required standards must be routinely checked. With chemical analyses, standards may be analysed together with the samples. By so doing errors could be detected and corrected. A periodic interlaboratory analysis to compare results in case of doubts is also helpful as this will help to ensure the reproducibility of results. Managers of research laboratories must ensure the implementation and enforcement of these measures.

Furthermore, research institutions must establish and allocate more training and teaching resources particularly for early career researchers on the scientific research process including the experimental design, methods as well as analysis, the management and publication of data [7]. However, the training should not be limited to early career scientists alone but to technicians, mid-career, and senior researchers. Since much of the crisis can also be traced to mid-career and senior researchers engaging in questionable practices and shortcuts out of habits, because they have benefited from these over their longer careers. Hence, training should be provided across board and can be incorporated in the mentorship training of young scientists, tenure positions or funding schemes which will be crucial in helping scientists understand the ethical implications of their work. This allows researchers to dedicate and focus on the essential details of their study and eliminate research bias [2, 7]. In line with this, the United State National Institute of Health developed and implemented a mandatory training course in promoting reproducibility and transparency of research findings with special focus on good experimental design for its fellows [3]. Also, extensive platform must be provided to train researchers on the implications of research integrity as well as avenues to discuss challenges. Research institutions must ensure the transparency and accessibility of research by incentivizing researchers who promote open science through the publication of open data of their research findings. These incentives could include long term research contracts, promotion, assigning tenure positions and providing easily accessible research grants. This will ensure a clear and focussed direction in doing better and productive research, since withholding important data that may be timely and innovative in order to secure promotion, tenure position or funds to sustain their career could hinder innovative progress. Additionally, institutions must endeavour to have and implement policies on good scientific practice with special focus on reproducibility. This includes putting in place measures that allows research employees to submit raw data upon request, which promotes transparency. Over-reliance on publication in high impact journals to assign tenure positions and promote researchers to a higher career level must be reduced. Instead, institutions must establish standard structures focusing on research integrity and quality in assigning these positions. In addition to providing resourceful tools and materials such as online storage servers, electronic laboratory notebook, research institutions must reward, promote, and provide guidelines on the publication of negative results [2, 7].

Finally, publishers must promote the publication of unexpected data and findings. This is very important as within the scientific fraternity so call novel results are awarded by fast publication whereas those which have so called negative results are not published. Also, grant awarding institutions should be open to giving different teams resources for same work. In so doing one team becomes a check on the other. However, this can be very costly and difficult but the need to ultimately save more lives outweighs the cost when for instance many lives depend on research findings that could be translated into new therapeutic findings like breakthrough drugs. Giving different teams resources for same work, could also deny other researchers the opportunity to explore new areas of research and prevent the possibility of diversity in exploring new frontiers of research.

**Fig. 1:**
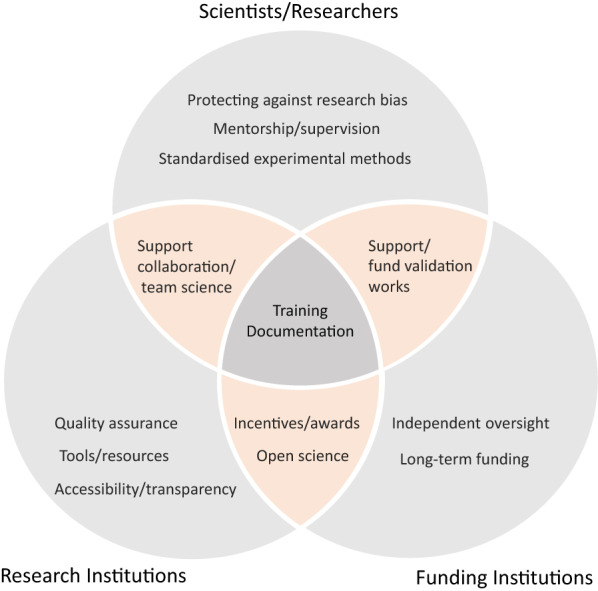
Potential responsibilities of each stakeholder and their intersectionality

Research integrity and reproducibility is very crucial. Here we summarise in a diagram, the individual and joint roles of different stakeholders in addressing the reproducibility crises (Fig. 1).

## Outlook

We all must be on the lookout and guard against any act that may undermine integrity of any research work. Going forward, postgraduates, postdocs, supervisors, technicians, laboratory managers and all who are involved in research needs to have compulsory and periodic courses on research integrity throughout their career and must be an integral part of their professional development. All hands must be involved in addressing this crisis. This will require the participation and contribution of all, such as scientists, institutions, and different stakeholders to promote reproducibility and preserve the integrity of research.

## Data Availability

Not applicable.
